# Enteral Nutrition Prescription in Children and Adults with Inflammatory Bowel Diseases: Gaps in Current Gastroenterology Practice in Saudi Arabia

**DOI:** 10.3390/nu15010232

**Published:** 2023-01-02

**Authors:** Sarah M. Ajabnoor, Atheer Attar, Noof BinJahlan, Nawal Almutairi, Shimaa Bashmail, Almoutaz Hashim, Alastair Forbes, Hani Jawa

**Affiliations:** 1 Clinical Nutrition Department, Faculty of Applied Medical Sciences, King Abdulaziz University, P.O. Box 80215, Jeddah 21589, Saudi Arabia; 2 National Nutrition Committee (NNC), Saudi Food and Drug Authority (Saudi FDA), Riyadh 13513, Saudi Arabia; 3 Department of Internal Medicine, University of Jeddah, Jeddah 21959, Saudi Arabia; 4 Institute of Clinical Medicine, University of Tartu, Tartu 51003, Estonia; 5 Department of Medicine, King Abdulaziz University, Jeddah 21589, Saudi Arabia

**Keywords:** inflammatory bowel disease, enteral nutrition, exclusive, gastroenterologist, practice, Crohn’s disease

## Abstract

Background: Evidence for the effectiveness of enteral nutrition (EN) for the management of patients with inflammatory bowel disease (IBD) is well-established. However, there is considerable global variation in EN practices. This study aimed to characterize the practices and perceptions of gastroenterologists regarding the use of EN in patients with IBD in one of the largest countries in the Gulf region. Methods: A cross-sectional study was conducted on pediatric and adult gastroenterologists working in Saudi Arabia who are involved in IBD management. A self-administered web-based survey was distributed via social media platforms and mailing lists of national gastroenterology societies. Results: A total of 80 gastroenterologists completed the survey. However, only 55 reported that they were currently practicing EN in any form. EN was mostly indicated by gastroenterologists who “sometimes” recommend EN for: the prevention and correction of undernutrition (50.9%), preoperative optimization (50.9%), and the induction of remission in patients with active and long-standing CD (36.4%), at initial diagnosis (34.5%), during the management of complications (61.8%), and after failing to respond to pharmacological therapy (58.2%). Exclusive enteral nutrition (EEN) is regularly recommended by 14.5% of gastroenterologists. The prescription of EEN was significantly associated with the pediatric profession (*p* < 0.01), IBD specialty (*p* < 0.05), level of nutrition education during training (*p* < 0.01), and previous training in a unit with regular EN use (*p* < 0.01). The most reported barriers to using EN were patients’ lack of acceptance (73.8%) and poor adherence (65%). A lack of dietitian support and a lack of standardized protocols were also reported as barriers by many physicians. Pediatric gastroenterologists were more likely to use at least one assessment method to evaluate EN success. Conclusion: EN practices differ between gastroenterologists working in Saudi Arabia. Future EN protocols should be optimized to support both children and adults with IBD. Gastroenterology training programs should offer nutrition support-focused training to help physicians better utilize EN.

## 1. Introduction

The incidence of inflammatory bowel disease (IBD) has been increasing globally. Recent reports from both Western and Eastern countries found an increasing trend in the incidence of IBD (both ulcerative colitis (UC) and Crohn’s disease (CD)) [1]. In Saudi Arabia, only data concerning the incidence rates of IBD in children have been reported, which was 0.47/100,000 population, with a significantly increasing trend [2].

The main goal of IBD treatment is to induce clinical remission and mucosal healing [3]. Nutrition plays an important role in IBD treatment. EN is frequently used for the correction of malnutrition in IBD and, increasingly, it is used as a primary treatment for CD. Exclusive enteral nutrition (EEN) is the most promising dietary intervention in CD as it provides all of the patient’s nutritional requirements through a liquid formula—delivered either orally or via a feeding tube—for a consistent period of time [4,5]. The efficacy of EEN in inducing mucosal healing in IBD is more pronounced than steroid therapy, particularly in pediatric patients [4,6,7]. However, at least partly due to poor compliance, EEN is not frequently used by adult patients and current evidence is not conclusive in supporting its efficacy in these older patients [6]. Conversely, partial enteral nutrition (PEN), which provides up to 50% of nutritional requirements through specialized enteral feeds in addition to the consumption of regular or specially designed meals orally, has been reported to achieve better tolerance [8]. Nonetheless, EEN remains superior to PEN in inducing remission. PEN might help most in maintaining remission in IBD; however, further evidence of efficacy is warranted [9].

There is considerable international variation in the current practice and perception of the use of EN in the IBD population. Only a few international cross-sectional studies have investigated the practice of EN; the majority of these studies targeted pediatric gastroenterologists [10,11,12,13], while one study in New Zealand assessed the practice of both pediatric and adult gastroenterologists [14]. A higher utilization of EN in pediatric IBD patients has been reported from European countries, Australia, New Zealand, and Canada [10,11,12,13]. A survey of Japanese physicians also reported a high rate of EN practice in children with CD [12]. Moreover, these studies were able to identify factors and barriers in the healthcare system that may have contributed to the variation in EN practice and outcomes in IBD [10,11,12,13]. However, the practice of EN for IBD in the Middle East is poorly reported. A variation in adherence to the international clinical practice guidelines by pediatric gastroenterologists working in Saudi Arabia was reported by one study, which investigated the general practice of different diagnostic and medical therapeutic interventions [15]. More investigation is needed to characterize EN practices in IBD in the Gulf region. The present study aimed to investigate the current practices and perceptions of both adult and pediatric gastroenterologists for the use of EN (either exclusively or partially) in the management of patients with IBD in Saudi Arabia.

## 2. Methods

### 2.1. Study Design

This is a cross-sectional study that used a self-administered web-based survey via the Google Forms platform. Gastroenterologists who are members of national societies (i.e., Saudi Gastroenterology Association [SGA] or the Saudi Society of Pediatric Gastroenterology, Hepatology, and Nutrition [SASPGHAN]) were recruited via a personal online invitation, in addition to snowball sampling through social media. The recruitment period was between January and March 2021. A reminder invitation was sent 3 to 4 weeks after the initial invitation if there was no response. Our inclusion criteria included all currently practicing gastroenterologists working in Saudi Arabia and managing patients with IBD. Physicians who did not specialize in gastroenterology were excluded. For the sample size calculation, the total number of physicians in Saudi Arabia specializing in gastroenterology was obtained from the latest statistical report published by the Ministry of Health (MOH) in 2019 [16]. With a total of 516 gastroenterologists, the required sample size was estimated to be 125 with a 80% confidence level, 5% margin of error, and a design effect of 1. The sample size was determined using the Epi InfoTM software (Epi Info 7.2.4.0, CDC, Atlanta, GA, USA). However, the number of IBD specialists in Saudi Arabia is not known, but international comparisons suggest that it is much less than 20% of all specialist gastroenterologists [17,18].

### 2.2. Questionnaire

The questionnaire was developed and adopted from previously published and validated surveys [10,11,13,19,20]. It consisted of four main sections: demographics, current use of any form of EN, detailed section for EEN practice and protocol, and opinions and perspectives toward the general use of EN in patients with IBD (Supplementary file S2). Clear definition of the different terms (i.e., EN, EEN, and PEN) were explained in the questionnaire. Face and content validity of the questionnaire was assessed using an expert panel consisting of two gastroenterologists and two clinical dietitians. The study investigators revised and resolved the questionnaire in line with the suggestions and feedback received.

### 2.3. Ethical Approval

This study was approved by the Applied Medical Sciences Research Ethical Committee at King Abdulaziz University in Jeddah, Saudi Arabia (no. FAMS-EC2020-0017). A brief description of our study’s purpose and statements regarding confidentiality and anonymity, in addition to informed consent, was included at the beginning of the survey.

### 2.4. Statistical Analysis

The data were analyzed using the Statistical Package for Social Sciences (SPSS) (version 20; IBM Corp., Armonk, NY, USA). Descriptive statistics of percentages and frequencies were used to describe categorical variables, whereas medians and interquartile ranges were used for continuous variables. Associations between categorical variables, such as gastroenterologists’ type of practice or gastroenterologists’ EEN prescribing status, and independent variables were assessed using a chi-square test. In addition, Fisher’s exact test was performed to assess associations between variables with smaller groups. *p*-values < 0.05 were considered statistically significant.

## 3. Results

### 3.1. Respondent Demographics

Among 516 physicians who were invited to participate, 103 responded to the survey, with 18 being excluded for reasons related to duplication (*n* = 6), disagreement about completing the survey (*n* = 2), not specializing in gastroenterology (*n* = 10), and being a GI fellow (*n* = 5). The final number of gastroenterologists included in the analysis was 80. The demographic characteristics of the participants are presented in Table 1. Most of the participants were men (77.5%). Thirty-six of the participants (45%) had been practicing for more than 10 years. The country of GI training for the majority of participants was Saudi Arabia (68.3%), followed by Canada (22.5%) and the USA (11.3%), and a few had completed their training in other countries (Australia, Jordan, UK, and elsewhere in Europe). Twenty-eight of the respondents (35%) identified themselves as IBD specialists or held an advanced fellowship in IBD, but this most likely indicates that our study has been responded to by at least a third of all IBD specialists in Saudi Arabia. Forty-three of the respondents (53.8%) rated their level of nutrition education during training as just adequate.

The proportion of gastroenterologists who had previously practiced in a unit where EN was regularly used (either exclusively or partially) was 41.3% (*n* = 33), while the proportion of all gastroenterologists (including adult and pediatric gastroenterologists) who reported that they were currently practicing any form of EN in IBD was 68.8% (*n* = 55).

### 3.2. Frequency of EN Recommendations

**Figure 1:**
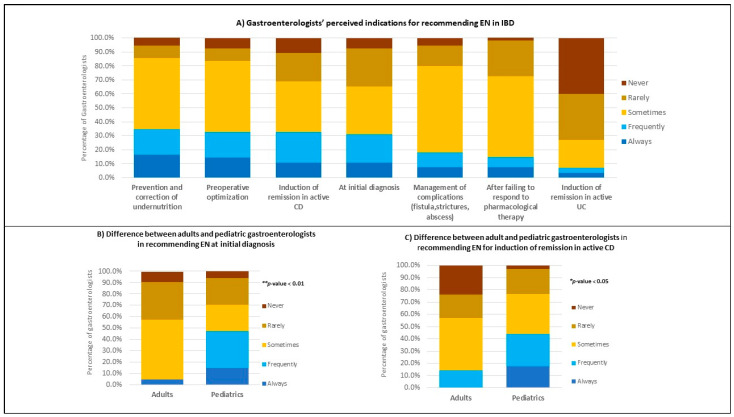
Indications for the use of any form of EN. (**A**) Indications reported by both adults and pediatric gastroenterologists (*n* = 55). (**B**) Percentage of adult (*n* = 21) versus pediatric (*n* = 34) gastroenterologists who are currently recommending EN at initial diagnosis. (**C**) Percentage of adult (*n* = 21) versus pediatric (*n* = 34) gastroenterologists who are currently recommending EN for the induction of remission in active CD. *p*-values indicate the statistical difference in proportions between groups (using Chi-square and Fisher’s exact tests).

The reported indications for EN use in any form are summarized in Figure 1A (detailed in Supplementary Table S1). A total of 55 gastroenterologists reported that they were currently practicing EN. Those respondents were asked to select one of the five options (never, rarely, sometimes, frequently, and always) to denote the frequency of their indications. The highest responses were indicated by gastroenterologists who “sometimes” recommend EN for: the prevention and correction of undernutrition (50.9%), preoperative optimization (50.9%), and the induction of remission in patients with active and long-standing CD (36.4%), at initial diagnosis for new patients (34.5%), during management of complications (61.8%), and after failure to respond to pharmacological therapy (i.e., biological, steroids, or immunosuppressant medications, etc.) (58.2%). However, to induce remission in active UC, many gastroenterologists (40%) reported that they never recommend EN.

A significant difference (*p* < 0.01) was observed between adult and pediatric gastroenterologists in their frequency of reported indications in Figure 1B. The percentage of pediatric gastroenterologists who frequently recommend EN at initial diagnosis for new patients was higher (32.4%) than adult gastroenterologists (0%).

### 3.3. Characteristics of EN Practices (Feeding Route, Formula Type, Methods of Evaluating Treatment Success, and Frequency)

**Figure 2:**
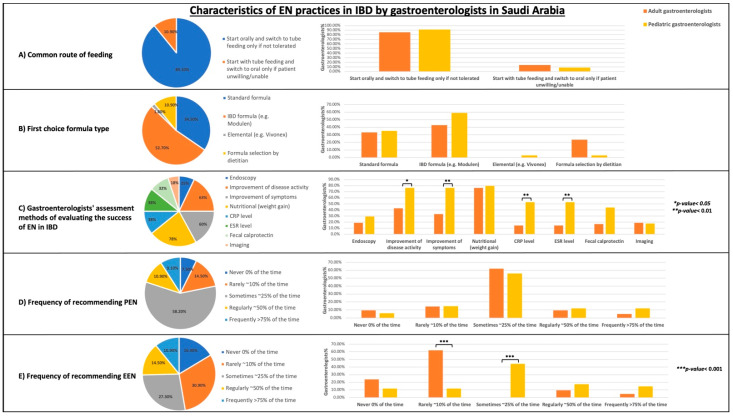
Characteristics of practicing EN in any form. Results are presented in percentages. All gastroenterologists (*n* = 55), adults (*n* = 21), pediatric (*n* = 34). (**A**) Common routes of EN feeding. (**B**) First choice formula type. (**C**) Methods of evaluating EN treatment success in IBD (total percentages do not add up to be 100% because this is a multiple response variable). (**D**) Frequency of partial enteral nutrition (PEN) practice. (**E**) Frequency of exclusive enteral nutrition (EEN) practice. *p*-values indicate the statistical difference in proportions between groups (using Chi-square and Fisher’s exact tests).

Characteristics of using EN in any form for gastroenterologists who reported that they were currently practicing EN (*n* = 55) are summarized in Figure 2 (detailed in Supplementary Table S2). The most preferred route of enteral feeding by gastroenterologists was “start orally and switch to tube feeding only if not tolerated” (89.1%). Twenty-nine of the respondents (52.7%) chose an IBD formula (e.g., Modulen IBD, Nestle) as the first recommended choice for patients with IBD. A standard formula was recommended by 34.5% (*n* = 19). Only one respondent (1.8%) recommended an elemental formula. However, 10.9% (*n* = 6) of gastroenterologists practicing EN (mostly adult gastroenterologists) reported that formula selection was performed by a dietitian. No statistically significant difference was found between adult and pediatric gastroenterologists in terms of their preferred feeding route and formula type (Figure 2A,B).

The most frequently used methods for evaluating EN success were nutritional outcomes (i.e., weight gain), used by 78.2% (*n* = 43). This was followed by an improvement in disease activity and symptoms, which were used by 63.6% (*n* = 35) and 60% (*n* = 33), respectively. A smaller percentage of physicians used imaging (18.2%) and endoscopy (25.5%) to evaluate the success of EN treatment. C-reactive protein (CRP), erythrocyte sedimentation rate (ESR), and fecal calprotectin levels were used by 38.2% (*n* = 21), 38.2% (*n* = 21), and 32.7% (*n* = 18), respectively. Overall, pediatric gastroenterologists were more likely to use at least one assessment method to evaluate EN success than adult gastroenterologists. A statistically significant difference was found between adult and pediatric gastroenterologists when using the following assessment methods: improvement of disease activity (*p* < 0.05), improvement of symptoms (*p* < 0.01), CRP level (*p* < 0.01), and ESR level (*p* < 0.01) (Figure 2C).

PEN was regularly used (about 50% of the time) by 10.9% of respondents (*n* = 6). The majority of respondents (58.2%) sometimes recommended PEN (about 25% of the time). However, EEN is regularly recommended (about 50% of the time) by 14.5% of respondents (*n* = 8). The proportion of respondents who used EEN rarely or sometimes is 30.9% (*n* = 17) and 27.3% (*n* = 15), respectively.

### 3.4. Exclusive Enteral Nutrition Practices

Of the total number of gastroenterologists who reported that they were currently practicing EN in any form (*n* = 55), only 46 indicated that they were specifically recommending EEN at least 10% of the time for patients with IBD. The characteristics of the current EEN protocols applied by those gastroenterologists are summarized in Table 2. The median number of patients treated with EEN by gastroenterologists in the previous year was 2 (IQR = 0–15). The most commonly reported factors influencing EEN recommendations were disease location and behavior (69.6%), patient’s age (69.6%), and patient’s education and personality (65.2%). In addition, expertise of clinical dietitians was reported as a key factor affecting EEN practice by 58.7% (*n* = 27). EEN was prescribed by 41.3% (*n* = 19) for 2–4 weeks, by 21.7% (*n* = 10) for 4–6 weeks, and by 26.1% (*n* = 12) for 6–8 weeks. However, only 6.5% (*n* = 3) used it for >8 weeks. Fourteen physicians practicing EEN (30.4%) reported that they allow concurrent oral intake, with several different types of allowed foods being identified. Moreover, twenty-three physicians (50%) recommended returning to the patient’s previous diet after completing EEN treatment, whereas seventeen (37%) recommended an ongoing special diet such as the CDED or the low FODMAP. Only two physicians (4.3%) recommended a continuing high-protein diet.

### 3.5. Comparison between EEN-Prescribing and non-EEN-Prescribing Gastroenterologists

This study further assessed whether EEN prescriptions were associated with the demographic characteristics of the respondents (Table 3). Notably, the proportion of pediatric gastroenterologists was significantly higher in the EEN prescriber group (65%) than that in the non-EEN prescriber group (34%) (*p* < 0.01). A statistically significant association was found between IBD specialty and EEN prescription status (*p* < 0.05). The level of nutrition education during training was significantly associated with EEN prescription status (*p* < 0.01). The percentage of EEN prescribers was high (65%) for gastroenterologists who perceived themselves as having an adequate level of nutritional education. In addition, a significant association was observed between the previous training variable and EEN prescription status (*p* < 0.01). The percentage of EEN prescribers was high (54%) for participants who had previously trained in a unit with regular use of EN in IBD.

### 3.6. Perceptions and Opinions toward EN Use in Patients with IBD

All the respondents’ perceptions (*n* = 80) of patients’ willingness toward different nutritional therapies are summarized in Table 4. Only 10% (*n* = 8) of respondents reported that patients were likely to accept and comply with EEN therapy, while 23.8% (*n* = 19) reported that patients were likely to accept PEN (with or without exclusion diets). Regarding exclusion or modified diets (without enteral supplements), 43.8% (*n* = 35) of respondents were neutral about their patients’ compliance. No statistically significant difference was found between adult and pediatric gastroenterologists in the perception of patients’ willingness to use nutritional therapies.

The gastroenterologists’ perceptions of the benefits and barriers to prescribing EN in any form in patients with IBD are described in Table 5. The highly perceived benefits were improving nutritional status (75%), maintaining growth (68.8%), and inducing remission in newly diagnosed CD (68.8%). The benefits associated with being a steroid-sparing therapy, inducing remission in newly diagnosed CD, and mucosal healing were perceived by pediatric gastroenterologists as significantly greater than adult gastroenterologists (*p* < 0.01).

The barriers perceived to most affect the general use of EN were patients’ non-acceptance (73.8%) and poor adherence due to the palatability of the formula (65%). A lack of dietitian support and a lack of standardized protocols were also reported as barriers by 57.5% (*n* = 46) and 58.8% (*n* = 47), respectively. Seventeen reported cost as a barrier (21.2%) and only one respondent (1.2%) reported no barriers affecting EN practice. Adult gastroenterologists were more likely to perceive the lack of dietitians (*p* < 0.05) and standardized protocols (*p* < 0.001), as well as the formula cost (*p* < 0.05), as barriers influencing their EN practice compared to the perceptions of pediatric gastroenterologists. Other barriers (e.g., poor adherence and disruption of normal life) were perceived to be significantly greater by pediatric gastroenterologists (*p* < 0.05 and *p* < 0.01, respectively).

Furthermore, the future existence of national guidelines was reported by the majority of participants (71.2%) as a factor that might enhance their EN practice in patients with IBD. More evidence of efficacy was perceived to be a key factor by thirty-six participants (45%), which was significantly higher than that reported by adult gastroenterologists (*p* < 0.001). Only five participants (6.2%) reported that they had already believed in the benefits of EN in IBD.

## 4. Discussion

The results of this study initially described the practice and perception related to the use of all forms of EN in IBD by gastroenterologists working in Saudi Arabia, including indications, choice of formula, assessment methods, and barriers to the use of EN. Pediatric gastroenterologists are generally more exposed to EN within their practice and more aware of the evidence in support of EN. The results of this study were able to identify the differences in practices and attitudes toward EN use between pediatric and adult gastroenterologists. Another important finding involved detailing the many aspects of currently administered EEN protocols, which will provide helpful resources for the improvement of future national protocols for EEN use in the IBD population.

In this study, the most frequently reported indications for the use of EN were inducing remission in active CD, preventing and correcting undernutrition, and managing complications. Although EN is appropriate in UC, physicians rarely used EN in UC cases as a primary therapy. However, EN is generally safe and can be recommended for nutritional support in active UC in the absence of contraindications, rather than bowel rest and parenteral nutrition [5].

Although many studies compared the effect of different enteral formulas (elemental vs. non-elemental) in patients with CD, the current European Society for Clinical Nutrition and Metabolism (ESPEN) guidelines recommend employing a standard polymeric formula rather than other specific formulas due to a lack of superior evidence [5]. In the current study, nearly half the gastroenterologists reported the use of an IBD-specific polymeric formula (Modulen IBD, Nestle, Switzerland), while standard formulas were recommended by 34.3% of our respondents. In contrast, the use of elemental feed was only reported by a few participants. This could be due to the lesser palatability of the elemental formula. However, cultural factors could have an impact on the practice and acceptance of EN therapy, which may explain how Japanese physicians are able to be more reliant on the use of elemental formulas and provide evidence suggesting its greater effectiveness among Japanese patients [12]. In addition, administering the formula via tube feeding could help improve adherence to EEN, which is an approach seen in previous international surveys [11,13] and the current study. Having a feeding tube deliver the formula may ensure full delivery, but it does not guarantee tolerance.

Evidence from previous international surveys agree that improvement of clinical symptoms and nutritional status is the most important outcome measure to consider when evaluating EN success [10,13]. The assessment methods used to evaluate EN success reported in this survey are similar to those described by previous surveys [10,13]. However, our study found that pediatric gastroenterologists were more likely to assess the improvement of disease activity or overall symptoms as well as the levels of inflammatory markers (CRP and ESR). This might be related to the fact that these assessment methods are less invasive and more practical for regular monitoring than radiology and endoscopy.

EN provided exclusively is a well-proven and validated therapeutic intervention which has been recommended as a first-line therapy for the induction of remission in children with active CD [5]. Global variation in the regular use of EEN has been reported among pediatric gastroenterologists, which ranges from 95% to 12% [10,12,13,21]. This study reported a low rate (14.5%) of regular EEN utilization. These results corroborate earlier findings from a previous survey of SASPGHAN members, where only 19% of respondents regularly prescribed EEN to their patients [15]. The results of the current study, not surprisingly, found that pediatric gastroenterologists are more likely to recommend EEN than adult gastroenterologists. The latest British guidelines on IBD management in adults acknowledge inadequate evidence of EEN in adult patients with Crohn’s, but indicate that when tolerated, it can be effective for the induction of remission. Therefore, the guidelines recommend including EEN as an option when counseling patients on treatment and providing practical prescription guidance [22].

The optimal duration of EEN in patients with CD has not been well defined. In the current study, 41.3% of gastroenterologists prescribed EEN over a period of 2–4 weeks, whereas 26.1% prescribed EEN for 6–8 weeks. However, in adults, an expert working group utilizing the best available evidence proposed tailoring the EEN duration to clinical indications [23]. Another aspect of the widely varied EEN protocol is how some clinicians might allow oral intake of “cheats” during EEN. In this study, 30.4% of “EEN” prescribers reported that they allowed oral intake. According to previous surveys, the provision of non-nutritive food items (e.g., candy, gum, clear fluids, and flavoring agents) is a common practice, whereas the addition of solid foods or one specialized meal per day during EEN was less common [10,11]. Offering other types of oral intake during EEN could be explained by the different perceptions and attitudes of physicians toward understanding the mechanism of action of EEN. Until now, the mechanism of action of EEN in IBD has not been clearly defined. However, EEN is a well-established intervention for the induction of remission in patients with CD compared to that of PEN therapy, which is only supported by limited evidence of efficacy [9]. In terms of the type of diet recommended after ending the course of EEN, the findings of this study indicate that there are variations in the current practice. Previous surveys have reported similar inconsistencies in the practice of food reintroduction [10]. However, there is no strong evidence to support the reintroduction of any specific diet after EEN therapy. Nevertheless, gradual food reintroduction (over 2–3 weeks after EEN) has been adopted by many clinicians [24]. An earlier retrospective study found that allowing normal foods after EEN did not necessarily induce any symptoms of food intolerance in children with CD [25], whereas recent findings by Logan et al. indicated that such practices can rapidly induce subclinical inflammation with increasing levels of fecal calprotectin [26]. More research is needed to describe the optimal approach for food reintroduction after EEN.

The current study found that selected demographic characteristics of gastroenterologists influenced the practice of EEN prescription. A similar pattern was observed in North America, Australia, and New Zealand where the utilization of EEN was higher among physicians with earlier exposure to EEN during their previous training [10,13]. Training at different sites and applying for international fellowships should always be encouraged among gastroenterology trainees. Due to the active role of gastroenterologists in nutrition support, nutrition-focused training should be enhanced throughout gastroenterology training programs.

Similar to a previous international survey by Lawley et al., the largest barriers affecting EN use reported in this study were patients’ lack of acceptance and poor adherence due to the palatability of the formula [11]. However, additional barriers such as the lack of dietitian support and the lack of standardized protocol were reported by many respondents (primarily adult gastroenterologists). This can be explained by the fact that EN use in adults with IBD is mainly supported by pediatric evidence. The existence of national guidelines is very important and was considered a major factor influencing the enhancement of EN prescription by our study respondents. Developing a standardized EN protocol specifically for adults with IBD is needed, which can then be used in future trials in adults to gain more evidence of efficacy. Only a few respondents indicated that cost was a barrier, which could be related to the healthcare system in Saudi Arabia where EN costs were typically covered (as in the UK), in contrast to the limited reimbursement of EN in the USA and Canada where cost was a major barrier [11].

This is the first study to describe the variations of and barriers to using EN in patients with IBD in the Middle East. In Saudi Arabia, only one study by Al-Sarkhy [15] investigated the variation in IBD care in children; however, this study did not characterize EN practices. Moreover, this study was limited by the small sample size (*n* = 37) [15], whereas the current study included a relatively larger sample size (*n* = 80). To our knowledge, the current study is the first to compare the EN practice of adult and pediatric gastroenterologists. This will provide key insights for the optimization of EN care in adults with IBD.

Limitations of this study include a selection bias. The survey was only distributed to gastroenterologists who were members of the SGA and SASPGHAN societies who opted to receive an electronic invitation from the society president or key members or follow the social media networks. Therefore, the study participants may not be completely representative of the gastroenterologists who are managing patients with IBD. Additionally, we were not able to come to a clear conclusion regarding perceived benefits and barriers for each type of EN treatment (EEN or PEN) by the respondents. This is because participants reported their perception of the benefits and barriers of the general use of EN only.

## 5. Conclusions

Overall, this study described the current practices and perceptions of both adult and pediatric gastroenterologists in the use of EN in IBD. This study will enable gastroenterologists and other IBD health care providers to focus on the identified practice gaps to develop a standardized protocol for EN use in children and adults with IBD, which will ultimately enhance patient care. Future survey-based studies targeting IBD patients and their families are still needed to understand the type of support they need during EN therapy. Finally, the findings of this study helped us understand the emerging need for nutrition support training in gastroenterology training programs in Saudi Arabia.

## Figures and Tables

**Table 1:** Participants’ demographic data (*n* = 80).

Demographic Variables	% (n)
**Gender**	
Male	77.5% (62)
Female	22.5% (18)
**Nationality**	
Saudi	88.8% (71)
Non-Saudi	11.3% (9)
**Region**	
Makkah	38.8% (31)
Madinah	2.5% (2)
Riyadh	30% (24)
Eastern Province	11.3% (9)
Asir	10% (8)
Najran	7.5% (6)
**Professional position**	
Adult gastroenterologist	48.8% (39)
Pediatric gastroenterologist	51.2% (41)
**Years of practice**	
<5 years	35% (28)
6–10 years	20% (16)
>10 years	45% (36)
**Practice setting ^$^**	
University teaching hospitals	27.5% (22)
Ministry of Health hospitals	37.5% (30)
Specialized hospitals	13.8% (11)
Military hospitals	23.8% (19)
National guard hospitals	5% (4)
Medical cities	2.5% (2)
Private medical centers	8.8% (7)
Other governmental institutions	6.3% (5)
**Placement country of GI training ^$^**	
Saudi Arabia	66.3% (53)
Canada	22.5% (18)
US	11.3% (9)
UK	2.5% (2)
Others (Germany, France, Australia, and Jordan)	6.3% (5)
**IBD specialty**	
IBD specialist	35% (28)
Non-IBD specialist	65% (52)
**Level of nutrition education during GI training**	
Inadequate	33.8% (27)
Just adequate	53.8% (43)
Excellent	12.5% (10)
**Previous training in a unit with regular use of EN in IBD**	
Yes	41.3% (33)
No	58.8% (47)
**Currently practicing EN in IBD**	
Yes	68.8% (55)
No	31.3% (25)

Percentages have been rounded and may not total to 100%. ^$^ Percentages do not add up to be 100% because this is a multiple response variable (participants selected more than one option).

**Table 2:** EEN practices and protocol characteristics (*n* = 46).

Variable	*%* (*n*)
Number of patients treated with EEN in the previous year *	2 (0–15)
**Factors affecting EEN recommendation ^$^**	
Patient’s age	69.6% (32)
Patient’s education and personality	65.2% (30)
Expertise of clinical dietitian	58.7% (27)
Disease location and behaviour	69.6% (32)
Cost of enteral nutrition	30.4% (14)
Other	13% (6)
**Duration of EEN**	
<2 weeks	4.3% (2)
2–4 weeks	41.3% (19)
4–6 weeks	21.7% (10)
6–8 weeks	26.1% (12)
>8 weeks	6.5% (3)
**Allowing oral intake when recommending EEN**	
Yes	30.4% (14)
No	63% (29)
I do not know	6.5% (3)
**Type of allowed oral intake while on EEN**	
Water only	6.5% (3)
Special foods (i.e., low fiber, liquid/soft, or any foods that do not irritate the bowel such as strawberries, chocolates, etc.)	6.5% (3)
Regular foods (or any kind of food for pleasure)	4.3% (2)
High protein/high calorie foods	10.9% (5)
One type of food allowed	2.2% (1)
I do not know	2.2% (1)
Skipped question	67.4 (31)
**Diet after EEN**	
Patient’s previous diet	50% (23)
Special diets (i.e., Crohn’s disease elimination diet, low FODMAP)	37% (17)
Other (i.e., high-protein diet as tolerated)	4.3% (2)
I do not know	8.7% (4)

Data are expressed as a median (range) for continues variables and as a percentage (frequency) for categorical data. * (*n* = 37) for gastroenterologists who were able to provide either actual or estimate numbers for patients. ^$^ Percentages do not add up to be 100% because this is a multiple response variable (participants selected more than one option). FODMAP: fermentable oligo-, di-, and monosaccharides, and polyols.

**Table 3:** Comparison between the demographic characteristics of EEN-prescribing and non-EEN-prescribing gastroenterologists (*n* = 80).

Demographic Characteristics	EEN Prescribers (*n* = 46) % (*n*)	Non-EEN Prescribers (*n* = 34) % (*n*)	*p*-Value *
**Gender** Male Female	83% (38) 17% (8)	71% (24) 29% (10)	0.203
**Nationality** Saudi Non-Saudi	87% (40) 13% (6)	91% (31) 9% (3)	0.555
**Region** Makkah Madinah Riyadh Eastern Province Asir Najran Other	41% (19) 2% (1) 35% (16) 6.5% (3) 6.5% (3) 9% (4) 0% (0)	35% (12) 3% (1) 23% (8) 18% (6) 15% (5) 6% (2) 0% (0)	0.451
**Professional position** Adult gastroenterologists Pediatric gastroenterologist	35% (16) 65% (30)	68% (23) 34% (11)	0.004
**Years of practice** <5 years 6–10 years >10 years	28.3% (13) 17.4% (8) 54.3% (25)	44% (15) 24% (8) 32% (11)	0.144
**Placement of GI training in Saudi Arabia** Yes No	67% (31) 33% (15)	65% (22) 35% (12)	0.802
**Placement of GI training in North America (Canada and US)** Yes No	24% (11) 76% (35)	35% (12) 65% (22)	0.266
**Placement of GI training in UK, Germany, and France** Yes No	9% (4) 91% (42)	9% (3) 91% (31)	0.984
**IBD specialty** IBD specialist Non-IBD specialist	46% (21) 54% (25)	21% (7) 79% (27)	0.020
**Level of nutrition education during GI training** Inadequate Just adequate Excellent	20% (9) 65% (30) 15% (7)	53% (18) 38% (13) 9% (3)	0.008
**Previous training in a unit with regular use of EN in IBD** Yes No	54% (25) 46% (21)	24% (8) 76% (26)	0.006

* *p*-values indicate the statistical difference in proportions between groups (using Chi-square analysis).

**Table 4:** Gastroenterologists’ perception of patients’ willingness toward different nutritional therapies (*n* = 80).

Type of Nutritional Therapy	Responses % (*n*)				
	Extremely Unlikely	Not Likely	Neutral	Likely	Extremely Likely
**Exclusive enteral nutrition**					
Adult gastroenterologists (*n* = 39)	25.6% (10)	46.2% (18)	20.5% (8)	5.1% (2)	2.6% (1)
Pediatric gastroenterologists (*n* = 41)	13.7% (13)	34.2% (14)	19.5% (8)	14.6% (6)	0.0% (0)
Total	28.8% (23)	40% (32)	20% (16)	10% (8)	1.3% (1)
*p*-value *	0.428				
**Partial enteral nutrition with or without exclusion diets ^$^**					
Adult gastroenterologists (*n* = 39)	2.6% (1)	30.8% (12)	41% (16)	23.1% (9)	2.6% (1)
Pediatric gastroenterologists (*n* = 41)	4.9% (2)	31.7% (13)	34.1% (14)	24.4% (10)	4.9% (2)
Total	3.8% (3)	31.3% (25)	37.5% (30)	23.8% (19)	3.8% (3)
*p*-value *	0.933				
**Exclusion or modified diets alone without enteral supplements**					
Adult gastroenterologists (*n* = 39)	12.8% (5)	23.1% (9)	33.3% (13)	20.5% (8)	10.3% (4)
Pediatric gastroenterologists (*n* = 41)	2.4% (1)	19.5% (8)	53.7% (22)	19.5% (8)	4.9% (2)
Total	7.5% (6)	21.3% (17)	43.8% (35)	20% (16)	7.5% (6)
*p*-value *	0.226				

* *p*-values indicate the statistical difference in proportions between groups (using Chi-square analysis). Exclusion diet: a diet that excludes products known to have a pro-inflammatory effect on the intestinal mucosa (e.g., dairy products, animal fats, emulsifiers, and processed foods); Modified diets are special diets like gluten-free, low-fat, or low FODMAP diets.

**Table 5:** Gastroenterologists’ perception of benefits of and barriers to prescribing any form of EN in IBD.

Variables	Adult Gastroenterologists (*n* = 39) % (*n*)	Pediatric Gastroenterologists (*n* = 41) % (*n*)	Total (*n* = 80) % (*n*)	*p*-Value *
**Benefits of EN in IBD**				
Steroid-sparing	33.3% (13)	65.9% (27)	50% (40)	0.004
Inducing remission in newly diagnosed CD	46.2% (18)	90.2% (37)	68.8% (55)	0
Inducing remission in long-standing CD	25.6% (10)	39% (16)	32.5% (26)	0.201
Inducing remission in active UC	20.5% (8)	24.4% (10)	22.5% (18)	0.678
Maintaining remission	38.5% (15)	41.5% (17)	40% (32)	0.784
Improving nutritional status	71.8% (28)	78% (32)	75% (60)	0.518
Optimizing pre-operative nutritional status	64.1% (25)	63.4% (26)	63.7% (51)	0.949
Maintaining growth	64.1% (25)	73.2% (30)	68.8% (55)	0.382
Mucosal healing	30.8% (12)	61% (25)	46.2% (37)	0.007
Improving quality of life	41% (16)	46.3% (19)	43.8% (35)	0.632
I do not think it is effective	2.6% (1)	0% (0)	1.2% (1)	0.487
Other (i.e., effective in pediatrics)	5.1% (2)	0% (0)	2.5% (2)	0.234
**Barriers affecting the use of EN in IBD**				
Patient’s unacceptance	64.1% (25)	82.9% (34)	73.8% (59)	0.056
Patient’s poor adherence due to palatability	53.8% (21)	75.6% (31)	65% (52)	0.041
Lack of dietitian support	71.8% (28)	43.9% (18)	57.5% (46)	0.012
Lack of standardized protocol	82.1% (32)	36.6% (15)	58.8% (47)	0
Too costly	30.8% (12)	12.2% (5)	21.2% (17)	0.042
Disruption of normal life	33.3% (13)	63.4% (26)	48.8% (39)	0.007
No barriers	0% (0)	2.4% (1)	1.2% (1)	1
**Factors might enhance EN prescription**				
More evidence of efficacy	66.7% (26)	24.4% (10)	45% (36)	0
Existence of national guidelines for practice	71.8% (28)	70.7% (29)	71.2% (57)	0.916
More understanding of the mechanism	28.2% (11)	31.7% (13)	30% (24)	0.733
Patient and family acceptance and awareness	0% (0)	7.3% (3)	3.8% (3)	0.241
Better and cheaper enteral formulas	2.6% (1)	2.4% (1)	2.5% (2)	1
I already believe in the benefits of EN	0% (0)	12.2% (5)	6.2% (5)	0.055

Percentages do not add up to be 100% because this is a multiple response variable (participants selected more than one option). * *p*-values indicate the statistical difference in proportions between groups (using Chi-square and Fisher’s exact tests).

## Data Availability

The data that support the results of this study are available upon request from the corresponding author.

## Supplementary Materials

The following supporting information can be downloaded at: https://www.mdpi.com/article/10.3390/nu15010232/s1, Table S1: Indications for using EN reported by gastroenterologists who are currently recommending EN in patients with IBD (n = 55); Table S2: Characteristics of EN practices carried by gastroenterologist who are currently recommending EN in patients with IBD. Supplementary file S2: Questionnaire for the study.
