# Supplementary material for: Enteral Nutrition Prescription in Children and Adults with Inflammatory Bowel Diseases: Gaps in Current Gastroenterology Practice in Saudi Arabia
Source: Nutrients. 2023 Jan 2;15(1):232. doi: 10.3390/nu15010232 (PMC9824199; doi:10.3390/nu15010232)
Supplement: Supplementary file 1 [file nutrients-15-00232-s001.zip › EN in IBD study_Final Supplementary File S2 Survey.pdf]

Questionnaire for the study: “The Current Status of Enteral Nutrition Practice in Inflammatory Bowel Diseases in Saudi Arabia”

<u>Section 1: Demographics</u>

1. What is your gender?
   - ◻ Male
   - ◻ Female

2. What is your nationality?
   - ◻ Saudi
   - ◻ Non-Saudi

3. Which region do you practice in?
   - ◻ Makkah
   - ◻ Madinah
   - ◻ Riyadh
   - ◻ Al-Qassim
   - ◻ Eastern Province
   - ◻ Asir
   - ◻ Al-Bahah
   - ◻ Tabuk
   - ◻ Northern Borders
   - ◻ Jawf
   - ◻ Hail
   - ◻ Jizan
   - ◻ Najran

4. What position do you currently hold in your practice?
   - ◻ Consultant adult gastroenterologist
   - ◻ Consultant pediatric gastroenterologist
   - ◻ Specialist adult gastroenterologist
   - ◻ Specialist pediatric gastroenterologist
   - ◻ Others (Specify: ………………………)

5. For how long have you been practicing?
   - □ <5 years
   - □ 6- 10 years
   - □ >10 years

6. Specify your type of practice setting?
   - □ University teaching hospitals
   - □ Ministry of Health (MOH) hospitals
   - □ Specialized hospitals (King Faisal Specialist Hospital and Research Centre)
   - □ Military Hospitals
   - □ National guard hospitals
   - □ Medical cities
   - □ Private medical centers
   - □ Other (Specify: ………………………)

7. Specify the country where you had your gastroenterology training?
   - □ Saudi Arabia
   - □ Canada
   - □ US
   - □ UK
   - □ Other (Specify: ………………………)

8. Do you hold an advanced fellowship in IBD or do you identify yourself as an IBD specialist?
   - □ Yes
   - □ No

9. How would you rate the level of nutrition education during your gastroenterology training?
   - □ Excellent
   - □ Just adequate
   - □ Inadequate

10. Have you previously trained in/worked in a unit where enteral nutrition was used regularly in patients with IBD?
    - □ Yes
    - □ No

<u>Section 2: Current Practice of Enteral Nutrition in Patients with IBD</u>

11. How often are you recommending any form of Enteral Nutrition in your patient population with IBD for the following indications?
    <u>(Enteral Nutrition can be provided orally or via the use of enteral tube feeding to deliver a specially-designed liquid formula)</u>

	Never	Rarely	Sometimes	Frequently	Always
At initial diagnosis	☐	☐	☐	☐	☐
After failing to respond to pharmacological therapy	☐	☐	☐	☐	☐
Prevention and correction of undernutrition	☐	☐	☐	☐	☐
Induction of remission in active Crohn's disease	☐	☐	☐	☐	☐
Induction of remission in active Ulcerative Colitis	☐	☐	☐	☐	☐
Preoperative optimization	☐	☐	☐	☐	☐
Management of complications (fistula, strictures, abscess)	☐	☐	☐	☐	☐

12. In your practice, what is the most preferable approach for choosing the route of enteral feeding in patients with IBD?
    - □ Always start oral; switch to tube feeding only if not tolerated
    - □ Always start tube feeding; switch to oral only if patient unwilling/unable

13. If you are involved in selecting formula type when recommending Enteral Nutrition for patients with IBD, what is usually your FIRST choice of enteral formula?
    - □ Standard polymeric (e.g. Ensure plus)
    - □ Polymeric formula with added growth factors marketed for IBD (e.g. Modulen IBD)
    - □ Elemental (e.g. Vital or Vivonex)
    - □ Semi-elemental (e.g. Peptamen)
    - □ Not involved (formula selection done by dietitian)

14. How do you evaluate the success of EN? (include all that apply)
    - □ Endoscopy
    - □ Improvement of clinical disease activity index such as CDAI or PDAI
    - □ Improvement of symptoms
    - □ Nutritional (weight gain)
    - □ CRP level
    - □ ESR level
    - □ Fecal calprotectin
    - □ Imaging
    - □ Other, (Specify: ……………………….)

15. How often do you recommend **Partial Enteral Nutrition (PEN)** in patients with IBD?
<u>(PEN means providing up to 50% of food intake through a specially-designed liquid formula)</u>
   - □ *Never 0% of the time*
   - □ *Rarely ~10% of the time*
   - □ *Sometimes ~25% of the time*
   - □ *Regularly ~50% of the time*
   - □ *Frequently >75% of the time*

16. How often do you recommend **Exclusive Enteral Nutrition (EEN)** in patients with IBD?
<u>(EEN means providing all of the dietary requirements of a patient through a specially-designed liquid formula delivered orally or via a feeding tube for a consistent period of time)</u>
   - □ *Never 0% of the time* <u>***(Move to section 3)***</u>
   - □ *Rarely ~10% of the time*
   - □ *Sometimes ~25% of the time*
   - □ *Regularly ~50% of the time*
   - □ *Frequently >75% of the time*

17. In the last year, how many patients with IBD received Exclusive Enteral Nutrition in your current practice?
   - □ *Specify the number and type of IBD:……………………………………..*
   - □ *Don't know*

18. If you answered question 17, was the answer based on your estimate or actual data?
   - □ Estimate
   - □ Actual data

19. In your practice which of the following factors you consider are important before recommending Exclusive Enteral Nutrition in IBD? (include all that apply)
   - □ Patient age
   - □ Patient education and personality
   - □ Expertise of clinical dietitian
   - □ Disease location and behaviour
   - □ Cost of enteral nutrition
   - □ Others (specify: …………….)

20. For how long do you <u>usually</u> prescribe Exclusive Enteral Nutrition in IBD?
   - □ < 2 weeks
   - □ 2-4 weeks
   - □ 4-6 weeks
   - □ 6-8 weeks
   - □ > 8 weeks

21. When recommending Exclusive Enteral Nutrition do you allow for any other oral intake?
    - ◻ No
    - ◻ Yes (specify what type of food items:…………………………)
    - ◻ I don't know

22. In your practice what type of diet do patient usually start AFTER Exclusive Enteral Nutrition course is over?
    - ◻ I don't know
    - ◻ Patient's previous diet
    - ◻ Specific diet (Crohn's disease elimination diet, low FODMAP)
    - ◻ Other (Specify: …..)

<u>Section 3: Opinions and Perspectives of Gastroenterologist of Using Enteral Nutrition In The Management of IBD</u>

23. In your opinion, how likely are patients with IBD willing to accept and comply with the following dietary therapies?

	Extremely unlikely	Not likely	Neutral	Likely	Extremely likely
Exclusive enteral nutrition	☐	☐	☐	☐	☐
Partial enteral nutrition with or without exclusion diets	☐	☐	☐	☐	☐
Exclusion or modified diets alone without enteral supplements	☐	☐	☐	☐	☐

24. To the best of your knowledge, what are proven benefits of Enteral Nutrition in IBD (include all that apply)
    - ◻ Steroid sparing
    - ◻ Inducing remission in newly diagnosed CD
    - ◻ Inducing remission in long-standing CD
    - ◻ Inducing remission in active UC
    - ◻ Maintaining remission
    - ◻ Improving nutritional status
    - ◻ Optimizing pre-operative nutritional status
    - ◻ Maintaining growth
    - ◻ Mucosal healing
    - ◻ Improving quality of life
    - ◻ I don't think it is effective as a therapeutic option
    - ◻ Others (Specify:………..)

25. In your opinion, what are the major barriers affecting your current practice of Enteral Nutrition in IBD? (include all that apply)
   - □ Patient unacceptance
   - □ Patient's poor adherence due to palatability
   - □ Lack of dietitian support
   - □ Lack of standardized protocol
   - □ Too costly
   - □ Disruption to normal life
   - □ None
   - □ Others (Specify: ………………...)

26. What would you wish to see before considering using Enteral Nutrition regularly in IBD? (include all that apply
   - □ More evidence of efficacy
   - □ National guidelines for practice
   - □ More understanding of the mechanism
   - □ Others (Specify: …………………..)

27. Your details:
   - □ I prefer to remain anonymous
   - □ I agree to be contacted and to provide additional information regarding my answers
     - Name:
     - Email:
     - Phone number:
