# Supplementary material for: Enteral Nutrition Prescription in Children and Adults with Inflammatory Bowel Diseases: Gaps in Current Gastroenterology Practice in Saudi Arabia
Source: Nutrients. 2023 Jan 2;15(1):232. doi: 10.3390/nu15010232 (PMC9824199; doi:10.3390/nu15010232)
Supplement: Supplementary file 1 [file nutrients-15-00232-s001.zip › EN in IBD study_Supplementary File S1 Tables and figures.pdf]

Supplementary Tables and Figures

***Table S1:** Indications for using EN reported by gastroenterologists who are currently recommending EN in patients with IBD (n= 55)*

Indications	Responses % (*n*)				
	Never	Rarely	Sometimes	Frequently	Always
**At initial diagnosis**					
Adult gastroenterologist	9.5% (2)	33.3% (7)	52.4% (11)	0% (0)	4.8% (1)
Paediatric gastroenterologists	5.9% (2)	23.5% (8)	23.5% (8)	32.4% (11)	14.7% (5)
Total	7.3% (4)	27.3% (15)	34.5% (19)	20% (11)	10.9% (6)
p-value[*]	*0.008*				
**After failing to respond to pharmacological therapy**					
Adult gastroenterologist	4.8% (1)	33.3% (7)	52.4% (11)	9.5% (2)	0% (0)
Paediatric gastroenterologists	0% (0)	20.6% (7)	65.6% (21)	5.9% (2)	11.8% (4)
Total	1.8% (1)	25.5% (14)	58.2% (32)	7.3 (4)	7.3% (4)
p-value[*]	*0.220*				
**Prevention and correction of undernutrition**					
Adult gastroenterologist	4.8% (1)	19% (4)	47.6% (10)	14.3% (3)	14.3% (3)
Paediatric gastroenterologists	5.9% (2)	2.9% (1)	52.9% (18)	20.6% (7)	17.6% (6)
Total	5.5% (3)	9.1% (5)	50.9% (28)	18.2% (10)	16.4% (9)
p-value[*]	*0.421*				
**Induction of remission in active CD**					
Adult gastroenterologist	23.8% (5)	19% (4)	42.9% (9)	14.3% (3)	0% (0)
Paediatric gastroenterologists	2.9% (1)	20.6% (7)	32.4 (11)	26.5% (9)	17.6% (6)
Total	10.9% (6)	20% (11)	36.4% (20)	21.8% (12)	10.9% (6)
p-value[*]	*0.036*				
**Induction of remission in active UC**					
Adult gastroenterologist	42.9% (9)	38.1% (8)	14.3% (3)	4.8% (1)	0% (0)
Paediatric gastroenterologists	38.2% (13)	29.4% (10)	23.5% (8)	2.9% (1)	5.9% (2)
Total	40% (22)	32.7% (18)	20% (11)	3.6% (2)	3.6% (2)
p-value[*]	*0.765*				
**Preoperative optimization**					
Adult gastroenterologist	0% (0)	4.8% (1)	57.1% (12)	23.8% (5)	14.3% (3)
Paediatric gastroenterologists	11.8% (4)	11.8% (4)	47.1% (16)	14.7% (5)	14.7% (5)
Total	7.3% (4)	9.1% (5)	50.9% (28)	18.2% (10)	14.5% (8)
p-value[*]	*0.474*				
**Management of complications (fistula, strictures, abscess)**					
Adult gastroenterologist	0% (0)	14.3% (3)	66.7% (14)	9.5% (2)	9.5% (2)
Paediatric gastroenterologists	8.8% (3)	14.7% (5)	58.8% (20)	11.8% (4)	5.9% (2)
Total	5.5% (3)	14.5% (8)	61.8% (34)	10.9% (6)	7.3% (4)
p-value[*]	*0.799*				

[*]*P*-value indicate the statistical difference in proportions between groups (using Fisher's exact tests

Table S2: Characteristics of EN practices carried by gastroenterologist who are currently recommending EN in patients with IBD

EN characteristic variables	Adult gastroenterologists (*n*= 21) % (*n*)	Pediatric gastroenterologists (*n*=34) % (*n*)	Total (*n*= 55) % (*n*)	*p-value*[*]
**Common route of feeding**				
Start orally and switch to tube feeding only if not tolerated	85.7% (18)	91.2% (31)	89.1% (49)	0.664
Start with tube feeding and switch to oral only if patient unwilling/unable	14.3% (3)	8.8% (3)	10.9% (6)	
**First Choice formula type**				
Standard formula	33.3% (7)	35.3% (12)	34.5% (19)	0.078
IBD formula (e.g. Modulen)	42.9% (9)	58.8% (20)	52.7% (29)	
Elemental (e.g. Vivonex)	0% (0)	2.9% (1)	1.8% (1)	
Semi-elemental (e.g. Peptamen)	0% (0)	0% (0)	0% (0)	
Formula selection by dietitian	23.8% (5)	2.9% (1)	10.9% (6)	
**Methods of evaluating EN success**[$]				
Endoscopy				
Improvement of clinical disease activity	19% (4)	29.4% (10)	25.5% (14)	0.391
index such as (CDAI)	42.9% (9)	76.5% (26)	63.6% (35)	0.012
Improvement of symptoms				
Nutritional (weight gain)	33.3% (7)	76.5% (26)	60% (33)	0.002
CRP level	76.2% (16)	79.4% (27)	78.2% (43)	1.000
ESR level	14.3% (3)	52.9% (18)	38.2% (21)	0.004
Faecal calprotectin	14.3% (3)	52.9% (18)	38.2% (21)	0.004
Imaging	14.3% (3)	44.1% (15)	32.7% (18)	0.22
	19% (4)	17.6% (6)	18.2% (10)	1.000
**Frequency of recommending PEN in IBD management**				
Never 0% of the time	9.5% (2)	5.9% (2)	7.3% (4)	0.949
Rarely ~10% of the time	14.3% (3)	14.7% (5)	14.5% (8)	
Sometimes ~25% of the time	61.9% (13)	55.9% (19)	58.2% (32)	
Regularly ~50% of the time	9.5% (2)	11.8% (4)	10.9% (6)	
Frequently >75% of the time	4.8% (1)	11.8% (4)	9.1% (5)	
**Frequency of recommending EEN in IBD management**				
Never 0% of the time	23.8% (5)	11.8% (4)	16.4% (9)	0.000
Rarely ~10% of the time	61.9% (13)	11.8% (4)	30.9% (17)	
Sometimes ~25% of the time	0% (0)	44.1% (15)	27.3% (15)	
Regularly ~50% of the time	9.5% (2)	17.6% (6)	14.5% (8)	
Frequently >75% of the time	4.8% (1)	14.7% (5)	10.9% (6)	

[*]*P*-value indicates the statistical difference in proportions between groups (using Chi-square and Fisher's exact tests)

[$]Percentages do not add to be 100% because this is a multiple response variable (participants selected more than one option)
